# Effectiveness of a two-tiered trauma team activation system at a level I trauma center

**DOI:** 10.1007/s00068-024-02644-2

**Published:** 2024-08-28

**Authors:** Jamela Abu-Aiada, Elchanan Quint, Daniel Dykman, David Czeiger, Gad Shaked

**Affiliations:** 1 Faculty of Health Sciences, Ben Gurion University of the Negev, Beer Sheva, Israel; 2 Department of General Surgery, Soroka University Medical Center, Ben- Gurion University, Beer Sheva, Israel; 3 Trauma Unit, Soroka University Medical Center, Beer Sheva, Israel

**Keywords:** Level I trauma center, Two-tiered trauma team activation, In-hospital triage, Overtriage, Undertriage

## Abstract

**Purpose:**

Many trauma patients who are transported to our level I trauma center have minor injuries that do not require full trauma team activation (FTTA). Thus, we implemented a two-tiered TTA system categorizing patients into red and yellow code alerts, indicating FTTA and Limited TTA (LTTA) requirements, respectively. This study aimed to assess the effectiveness of this triage tool by evaluating its diagnostic parameters (sensitivity, specificity, positive predictive value (PPV), negative predictive value (NPV), undertriage and overtriage) and comparing injury severity between the two groups.

**Methods:**

A retrospective cohort study of patients admitted to a Level I trauma center. Characteristics compared between the red and yellow code groups included demographics, injury severity, treatments, and hospital length of stay (LOS). Calculating the diagnostic parameters was based on Injury Severity Score (ISS) and the need for life-saving surgery or procedures.

**Results:**

Significant differences in injury severity indicators were observed between the two groups. Patients in the red code group had a higher ISS and New Injury Severity Score (NISS), a lower Glasgow Coma Score (GCS), Revised Trauma Score (RTS), and probability of survival. They had a longer hospital LOS, a higher Intensive Care Unit (ICU) admission rate and required more emergency operations. The Sensitivity of the triage tool was 85.2%, specificity was 55.6%, PPV was 74.2%, NPV was 71.5%, undertriage was 14.7%, and overtriage was 25.7%.

**Conclusion:**

The two-tiered TTA system effectively distinguish between patients with major trauma who need FTTA and patients with minor trauma who can be managed by LTTA.

## Introduction

Trauma is one of the leading causes of death worldwide with millions of people dying each year [1]. Trauma team activation (TTA) is a well-recognized standard of care to provide rapid stabilization of patients with time-critical, life-threatening injuries. TTA is associated with a substantial use of valuable hospital resources that may adversely impact upon the care of other patients if not carefully balanced [2]. Trauma triage is the process of categorizing trauma patients by injury severity and then allocating appropriate resources for care based on prioritization of their immediate surgical needs or by their likelihood to benefit from such resources [3].

The topic of trauma triage was divided into 3 subcategories: “field-triage” (choice of destination hospital), “hospital-triage” (level of TTA) and “triage-assessment” (assessment of the appropriateness of TTA as it relates to injury severity), matching the severity of a trauma patient’s injury with an appropriate level of hospital-triage to provide adequate resources remains a challenge [4]. Undertriage and overtriage rates are important quality indicators for trauma systems. The undertriage rate is often defined to capture the proportion of major trauma receiving suboptimal care. The overtriage rate is defined to capture the proportion of unnecessary use of hospital resource on minor trauma [5]. Undertriage can worsen patient outcome whereas overtriage mainly causes a burden for hospital resources as complete level one trauma care is utilized for patients without severe injuries [6]. An undertriage rate of less than 5% and an overtriage rate of less than 35% are often considered as acceptable according to the American College of Surgeons Committee on Trauma (ACS-COT) [5]. Efforts to reduce the impact of overtriage led to the development of tiered trauma activation systems. These systems seek to differentiate the severely injured patients who require a multispecialty trauma response, from the minimally injured patients, for whom an abbreviated trauma response will meet their medical needs [7].

For many years, our level I trauma center has followed the National guideline for the field triage of injured patients. According to these guidelines, field triage attempts to distinguish trauma patients that must be transported to the highest-level trauma center available from other trauma patients who do not require the highest level trauma center and can be treated in other facilities [8]. Our hospital serves as the sole major healthcare facility in southern Israel, encompassing over half of the state land area and serving a population of about one million people. Consequently, all trauma patients within our area are transported to our Level I trauma center, regardless of whether they require such a high level of care. This situation has resulted in unnecessary activations of trauma teams, high costs and inefficient resource utilization. To address this issue, we accompanied the field triage process with an in- hospital triage system. The in-hospital triage system is a two-tiered TTA system that aims to differentiate between major and minor trauma patients, activating the Full Trauma Team (FTT) only for major trauma cases and the Limited Trauma Team (LTT) for minor trauma cases. The aim of this study is to evaluate the effectiveness of this new triage system.

## Methods

### Design, settings and participants

A retrospective cohort study of trauma patients admitted to our Level I trauma center at Soroka University Medical Center, from February 1 to November 30, 2022.

### Data collection

The Chameleon medical record was used to gain information regarding the level of TTA for each patient. Data collected from the trauma registry were demographic information such as age and gender, type of injury, helicopter transport, transfer from another hospital, vital signs on admission, breathing method upon hospital entry, injury severity scoring systems (Injury Severity Score (ISS), New Injury Severity Score (NISS), Revised Trauma Score (RTS), Glasgow Coma Score (GCS) and Probability of survival calculated based on TRISS system), need for critical procedures in the emergency department (ED) including, endotracheal intubation, chest tube insertion, receiving blood products and requiring massive transfusion protocol (MTP), need for surgical intervention, direct transfer from the ED to the operating room (OR), need for specific emergency operations (craniotomy, laparotomy and thoracotomy), need for mechanical ventilation, Intensive Care Unit (ICU) admission, and hospital length of stay (LOS).

### Two-tiered trauma team activation protocol

Prior to the implementation of the two -tiered TTA system, TTA was based on pre-hospital triage performed by Emergency Medical Services (EMS) workers following field triage guidelines [8]. With the introduction of the two-tiered TTA system, EMS workers kept conducting the field triage to determine which patient required TTA. This information was then conveyed to a trauma triage nurse at the hospital. Based on the pre-hospital data and guidelines outlines in Table 1 (derived from the ACS-COT’s tiered trauma team activation system [9]), the trauma nurse performed in-hospital triage to assign the specific TTA level. These guidelines consist of physiologic, anatomic, and mechanistic criteria, and they indicate that patients meeting anatomical or physiological criteria require Full Trauma Team Activation (FTTA) while patients meeting only mechanistic criteria require Limited Trauma Team Activation (LTTA). If prehospital information is unavailable or insufficient a FTT is called, and in cases of self-transfer to the hospital, triage is performed by the triage nurse upon the patient’s arrival. Patients necessitating FTTA were designated as “red code,” while those requiring LTTA were designated as “yellow code”. Alerts for red and yellow codes were transmitted via the in-hospital trauma alert system, prompting notification and arrival of the appropriate trauma team. The composition of each trauma team, standby staff, and resources that should be available and accessible are presented in Table 2.

**Table 1:**
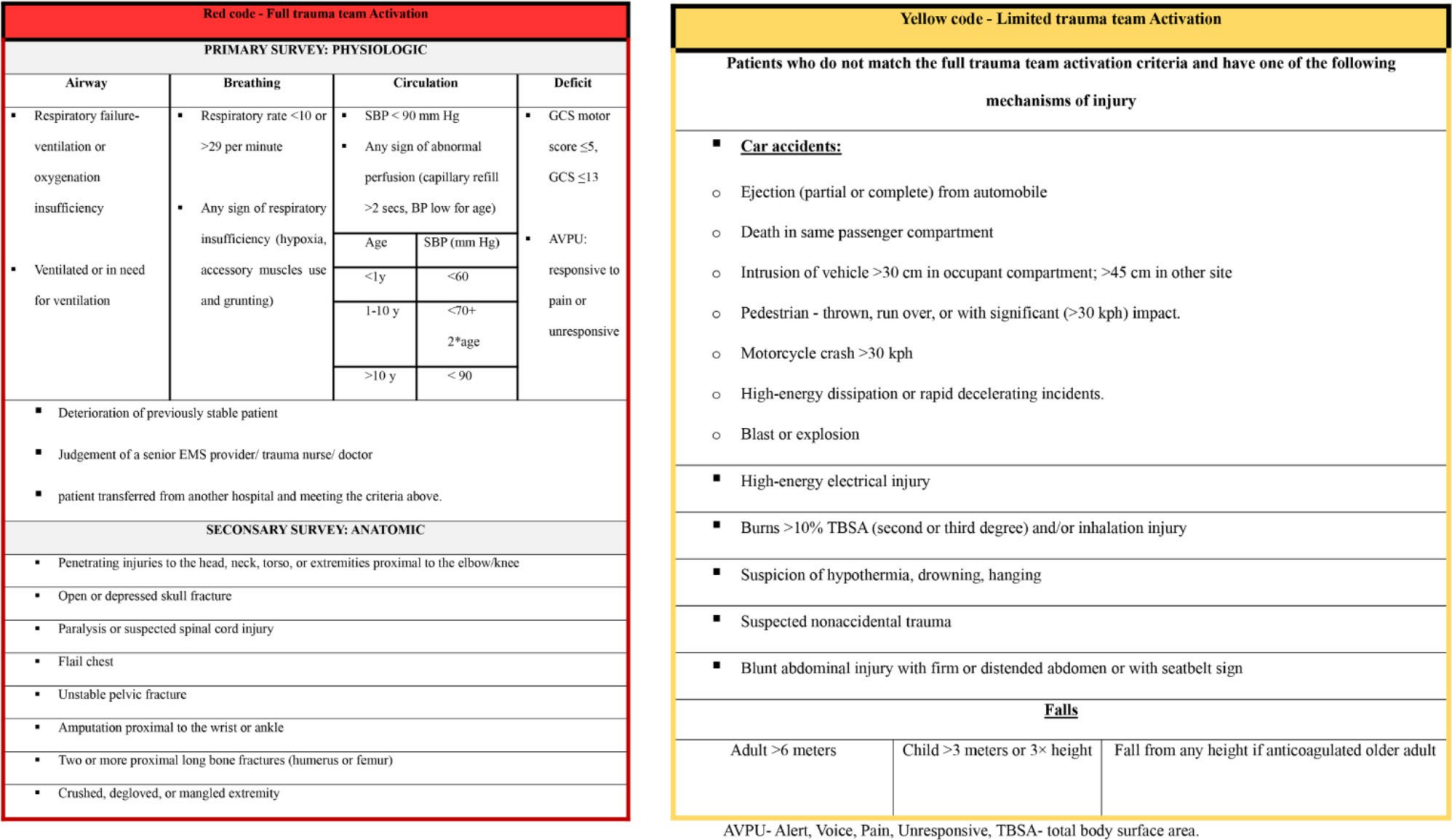
Two-tiered trauma team activation criteria

**Table 2:** Trauma team composition for red and yellow code alerts

Red code- Full trauma team Activation	Yellow code- Limited trauma team Activation
Senior surgical resident (PGY-4) Junior surgical resident ED doctor Involvement of children – pediatric surgeon and pediatric ICU doctor Anesthesiologist 2 ED nurses Radiology technician Phlebotomist Another surgical subspeciality may be necessary (orthopedic surgeon, neurosurgeon, cardiothoracic surgeon, etc.)	Senior surgical resident ED doctor Involvement of children – pediatric surgeon ED nurse
**Stand by staff**	
Attending Trauma surgeon The head of the trauma unit in special cases Blood bank and operating room coordinator must be notified.	Attending Trauma surgeon
**Resources that should be immediately available**	
Operating room CT Thromboelastography -TEG Massive transfusion protocol- MTP	CT

PGY-postgraduate year

### Data analysis

Statistical analyses were performed using SPSS software. The study population was described using descriptive statistics. Categorical variables are presented by absolute numbers, percentages and odds ratio and its 95% CI, and continuous variables by mean, standard deviation and mean difference and its 95% CI. To compare the two groups (red code vs. yellow code), the chi square test and fisher exact test were used to analyze categorical data. Mann-Whitney test was used to analyze ordinal data and unpaired t test was used to compare continuous data. A P-value of less than 0.05 was considered statistically significant. Major trauma was defined as any patient who meets at least one of the following criteria: ISS > 15, direct transfer from ED to the OR, and need for any critical procedure in the ED including, endotracheal intubation, chest tube insertion, and blood transfusion. The diagnostic parameters (sensitivity, specificity, positive predictive value (PPV), negative predictive value (NPV), undertriage and overtriage) were evaluated using this definition as a gold standard and based on the definitions presented in Table 3.

**Table 3:** Definitions of diagnostic parameters

	Major Trauma	Minor Trauma	Total
Red code	a	b	a + b
Yellow code	c	d	c + d
Total	a + c	b + d	N

Sensitivity = *a/ (a* + *c)*; specificity = *d/(b* + *d)*; PPV = *a/ (a* + *b)*; NPV = *d/ (c* + *d)* undertriage = *c/a + c;* overtriage = b*/ a + b*

## Results

### Study population

During the study period, 687 patients were admitted to our trauma center. Of these, 463 (67.4%) patients had documented levels of activation, with 319 (46.4%) labeled as red code, receiving FTTA, and 144 (21%) labeled as yellow code, receiving LTTA. Documentation on TTA level was not found in 224 (32.6%) cases.566 (82.4%) patients were male and 121 (17.6%) were female. The mean age was 28.6 (± 20.1) years. The most common type of injury was blunt injury in 501 (72.9%) patients followed by penetrating injury in 149 (21.7%) patients. The mean ISS was 12.7 (± 10.5), with 220 (32.1%) patients having an ISS > 15. In the ED, procedures included endotracheal intubation for 81 (11.8%) patients, chest tube insertion for 42 (6.1%), and initiation of MTP for 13 (1.9%) patients. Surgery after primary evaluation was required for 295 (42.9%) patients, with 111 (16.2%) being transferred directly from the ED to the OR. The mean hospital LOS was 7.7 (± 13.2) days. 202 (29.4%) patients were admitted to the ICU. For further details on the study population, please refer to Table 4. And for more information on the group of patients lacking a documented level of activation, please refer to Table 5.

**Table 4:** Characteristics of the study population

Variable	All patients (N = 687)
Age	28.6 ± 20.1
**Gender**	
Male	566 (82.4%)
Female	121(17.6%)
**Helicopter transport**	54(7.9%)
**Transfer from another hospital**	21(3.1%)
**Self-transfer to the hospital**	77 (11.2%)
**Type of code**	
Red code	319 (46.4%)
Yellow code	144 (21%)
None	224(32.6%)
**Injury Type**	
Blunt	501(72.9%)
Penetrating	149 (21.7%)
Crush	14 (2%)
Burn	18(2.6%)
Blast	5(0.7%)
**Vital signs upon admission**	
Heart rate	99 ± 25.5
Respiratory rate	20.4 ± 3.6
Systolic Blood Pressure	132.2 ± 26.6
Diastolic blood pressure	77.4 ± 16.5
Oxygen saturation	96.9 ± 5.2
**Breathing method upon admission**	
Spontaneous breathing	519(75.5%)
Simple face mask	113(16.4%)
Ambu face mask	12(1.7%)
LMA	3(0.4%)
Endotracheal tube	32(4.7%)
Unknown \ Other	8(1.1%)
**Critical Procedures in the ED**	
Chest tube	42(6.1%)
Endotracheal intubation	81(11.8%)
Receiving blood products	89(13%)
MTP	13(1.9%)
**Direct transfer from ED to OR**	111(16.2%)
**Emergency operations**	
Laparotomy	43(6.3%)
Craniotomy	19(2.8%)
Thoracotomy	5(0.7%)
**Need for surgical intervention**	295(42.9%)
**Need for mechanical ventilation**	139(20.2%)
**ICU Admission**	202(29.4%)
**Hospital LOS**	7.7 ± 13.2
**Trauma scoring systems**	
ISS	12.7 ± 10.5
ISS > 15	(32.1%)220
NISS	16 ± 13.7
GCS	13.7 ± 2.9
RTS	7.5 ± 0.8
Probability of survival	95.3
Probability of survival ≤ 95%	81(13.2%)

**Table 5:** Characteristics of the “no code” group

Variable	No code (N = 224)
**Age**	29.4 ± 20.9
**Gender**	
Male	181 (80.8%)
Female	43(19.2%)
**Injury Type**	
Blunt	142(63.4%)
Penetrating	65 (29%)
Crush	6 (2.7%)
Burn	10(4.5%)
Blast	1(0.4%)
**Critical Procedures in the ED**	
Chest tube	12(5.4%)
Endotracheal intubation	26(11.6%)
Receiving blood products	28 (12.5%)
MTP	4 (1.8%)
**Emergency operations**	
Laparotomy	14 (6.3%)
Craniotomy	5 (2.2%)
Thoracotomy	2 (0.9%)
**Need for surgical intervention**	86 (38.4%)
**ICU Admission**	51 (22.8%)
**Hospital LOS**	5.9 ± 8.7
**Trauma scoring systems**	
ISS	10.9 ± 9.4
ISS > 15	60 (26.8%)
NISS	13.6 ± 12.6
GCS	14.1 ± 2.5
RTS	7.5 ± 0.8
Probability of survival	95.7

### Comparing red code group vs. yellow code group

The comparison between the red code group and the yellow code group is presented in Table 6. The mean age was significantly lower in the red code group (26.1vs. 32.8, *p* < 0.001). Helicopter transportation was more frequent among patients in the red code group (11% vs. 4.9%, *p* = 0.034). A significantly lower proportion of patients in the red code group were able to breathe spontaneously upon arrival at the hospital (66.5% vs. 84.7%, *p* < 0.001). Upon admission, the red code group exhibited a significantly faster heart rate (101.9 vs. 92, *p* < 0.001), lower systolic blood pressure (SBP) (128 vs. 137.2, *p* < 0.001), and lower diastolic blood pressure (DBP) (76.1 vs. 81.2, *p* = 0.003). The necessity for critical procedures in the ED exhibited significant differences between the two groups. Patients in the red code group showed a higher demand for chest drainage (8.8% vs. 1.4%, *p* = 0.003), endotracheal intubation (14.7% vs. 5.6%, *p* = 0.005), and blood products transfusions (16.3% vs. 6.3%, *p* = 0.003).Additionally, a greater proportion of patients in the red code group necessitated emergency operations, defined as requiring emergent craniotomy, emergent laparotomy, or ED-thoracotomy (11.6% vs. 5.6%, *p* = 0.042).Trauma scoring systems highlighted disparities in injury severity between the two groups. The red code group demonstrated significantly higher NISS Score (18.8 vs. 13.5, *p* < 0.001) and ISS (14.9 vs. 10.6, *p* < 0.001). Furthermore, a larger percentage of patients in the red code group had an ISS greater than 15 (39.6% vs. 23.6%, *p* < 0.001), lower GCS (13.1 vs. 14.5, *p* < 0.001), RTS (7.3 vs. 7.6, *p* < 0.001), and probability of survival (93.9% vs. 97.6%, *p* < 0.001).Additionally, Patients in the red code group had a longer hospital LOS (9.7 vs. 5.7, *p* < 0.001), higher rates of ICU admission (37.9% vs. 20.8%, *p* < 0.001), and higher rates of mechanical ventilation (28.2% vs. 7.6%, *p* < 0.001).

**Table 6:** Comparison of the red code group against the yellow code group

Variable	Red code (FTTA) (N = 319)	Yellow code (LTTA) (N = 144)	P- value	Mean difference (CI 95%)	OR (CI 95%)
**Age**	26.1 ± 19.1	32.8 ± 20.4	0.001>	6.6(2.8–10.5)	
**Helicopter Transport**	35(11%)	7(4.9%)	0.034		2.4(1.04–5.5)
**Self – transfer to the hospital**	15 (4.7%)	4 (2.8%)	0.09		
**Vital signs on admission**					
Heart rate	101.9 ± 26.4	92 ± 20.4	< 0.001	9.9(5.4–14.4)	
Systolic Blood Pressure	128 ± 25.9	137.2 ± 22.1	< 0.001	9.1(4.1-14.07)	
Diastolic blood pressure	76.1 ± 17.4	81.2 ± 14.9	0.003	5.1 (1.7–8.4)	
**Breathing method on admission**			< 0.001		
Spontaneous breathing	212(66.5%)	122(84.7%)			
Simple face mask	70(21.9%)	22(15.3%)			
Ambu face mask	8(2.5%)	0(0%)			
LMA	2(0.6%)	0(0%)			
Endotracheal tube	25(7.8%)	0(0%)			
Unknown \ Other	1(0.3%)	0(0%)			
**ISS**	14.9 ± 12.1	10.6 ± 7.1	0.001>	4.7(2.4–9.2)	
**ISS > 15**	126(39.6%)	34(23.6%)	0.001>		2.1(1.3–3.3)
**NISS**	18.8 ± 15.2	13.5 ± 10.2	0.001>	5.2 (2.8–7.6)	
**GCS**	13.1 ± 3.6	14.5 ± 1.3	0.001>	1.4 (0.9–1.8)	
**RTS**	7.3 ± 1	7.6 ± 0.4	0.001>	0.3 (0.2–0.4)	
**Probability of survival**	93.9	97.6	0.001>	3.7 (1.6–5.8)	
**Probability of survival ≤ 95%**	49( 15.3%)	11(7.6%)	0.001>		
**Emergency operations**	37(11.6%)	8 (5.6%)	0.042		2.2 (1.01–4.9)
**Critical Procedures in the ED**					
Chest tube	28(8.8%)	2(1.4%)	0.003		6.8(1.6–29.0)
Endotracheal intubation	47(14.7%)	8(5.6%)	0.005		2.9 (1.3–6.3)
Receiving blood products	52(16.3%)	9(6.3%)	0.003		2.9(1.3–6.1)
**Need for mechanical ventilation**	90(28.2%)	11(7.6%)	0.001>		4.7(2.4–9.2)
**ICU Admission**	121(37.9%)	30(20.8%)	0.001>		2.3 (1.4–3.6)
**Hospital LOS**	9.7 ± 16.9	5.7 ± 7.9	0.001>	3.9(1.7–6.2)	

### Diagnostic parameters

According to the major trauma definition mentioned above, 278 patients suffered from a major trauma, of these patients, 237 patients received a red code, and 41 patients received a yellow code. 185 patients didn’t meet the major trauma criteria, 103 of them received a yellow code and 82 received a red code. Therefore, the sensitivity was 85.2% (237/278), specificity was 55.6% (103/185), PPV was 74.2% (237/319), NPV was 71.5% (103/144). Undertriage rate was 14.7% (41/278) and overtriage rate was 25.7% (82/319).

## Discussion

Trauma patients present varying degrees of injury severity, underscoring the importance of appropriate triage and transportation protocols. While it’s crucial for severely injured patients to receive expedited treatment through FTT, deploying FTTA for mildly injured patients may result in resource inefficiencies. To demonstrate the efficacy of a two-tiered TTA system, it’s imperative to establish its ability to differentiate between severe and mild injuries. The findings of this study suggest that our system meets these criteria successfully.

Our key findings align closely with previous research. Jenkins et al. [10] investigated the effectiveness of a two-tiered TTA system within a UK trauma center, while Davis et al. examined a similar system in an Australian context [7].In both studies, pre-hospital data played a pivotal role in determining the appropriate level of activation. The highest level of activation was initiated when physiological or anatomical criteria were met, while the lower level of activation was employed when only mechanism of injury criteria were present. Those two studies also demonstrated a correlation between the level of activation and the level of in-hospital care received by patients. Jenkins et al. found that patients with the highest level of activation required more endotracheal intubation, blood products, and emergency operations. They also exhibited higher ISS and a greater likelihood of ICU admission [10]. Similarly, Davis et al. noted a significantly extended LOS and a higher incidence of major trauma outcomes, defined as an ISS > 15, requirement for high dependency unit or ICU admission, urgent operative intervention, or in-hospital mortality [7]. Another study by Plaisier et al. implemented a two-tiered TTA system rooted in the ACS-COT recommendations, yielding results akin to our study. Their findings indicated that patients categorized under the highest level of activation demonstrated a higher ISS, an elevated need for emergency surgery, increased admission rates to the ICU, prolonged hospital LOS, and a higher occurrence of emergency procedures. [11]

Kouzminova et al. conducted a 10-year retrospective study to evaluate the safety and efficacy of a two-tiered TTA system, where trauma activations were categorized as “major” or “minor” based on pre-hospital information. Their findings indicated that the major trauma group exhibited a higher ISS, lower blood pressure upon presentation, lower GCS, a higher frequency of ED intubation, and increased ICU admission rates [12]. This retrospective analysis, spanning a decade of data, concluded that a two-tiered trauma activation system effectively identifies patients in need of a comprehensive trauma team response and may facilitate a more efficient utilization of trauma center resources.

In this study, we utilized the ISS and the need for life-saving surgery or procedures as a reference standard to evaluate the diagnostic parameters of the triage tool. Our findings demonstrated 41 cases (14.7%) of under-triage and 82 cases (25.7%) of over-triage. These results are in line with previous research on two-tiered TTA systems [6, 13–15]. 14.7% undertriage rate is not considerably higher than the maximum allowable undertriage rate of 5%, and a 25.7% over-triage rate falls within the permitted over-triage rate of 25 − 35% according to the definitions of ACS-COT. Considering that this is the initial phase of implementing a two-tiered trauma triage system in our hospital and that we are evaluating the performance of the triage tool during the first 10 months of its implementation, we assume that a 14.7% undertriage and a 25.7% overtriage rates are partly due to a learning curve, and we expect that these rates will improve even more as we gain more experience and better compliance and adherence with the new protocol. The sensitivity of the triage tool was 85.2%, while the specificity was 55.6%. In triage tools, sensitivity in detecting major trauma is considered more crucial than specificity due to the potential consequences of missing a significant injury. A lower sensitivity suggests that the test may underestimate severity, potentially failing to recognize many patients with major traumas. Thus, we are satisfied with a sensitivity of 85.2%, indicating that most patients with major trauma were identified and treated by FTT. Our results are consistent with the values of sensitivity and specificity reported in previous studies [6, 7, 16–19] as well as the values of PPV and NPV reported in similar research [16, 18, 20, 21].

### Limitations

Due to its retrospective nature, this study inherently has limitations, particularly concerning the total number of patients included. Notably, in 224 cases, documentation of the TTA level was lacking. As this information couldn’t be retrospectively obtained, patients with undocumented TTA levels were excluded from the study. However, data from 687 individuals admitted to our center and registered in the trauma registry were collected, with 463 of them having a verified TTA level. This sample size was deemed sufficient to represent the trauma population and conduct statistical analyses. Another limitation arises from the lack of consensus on the gold standard for calculating diagnostic parameters and evaluating the accuracy of triage tools [3]. Different studies have utilized various gold standards to assess the performance of their triage tools [3, 16].we chose to use the ISS as a reference standard along with other variables. ISS has been widely utilized in previous studies [3, 16, 22] and is recommended by the ACS-COT for defining major trauma and evaluating triage performance [23]. However, previous studies showed that ISS alone is insufficient for identifying major trauma patients requiring FTTA [24–27]. As a result, we included factors that suggest that a patient has had a major trauma and that a FTTA is needed in his situation, such as direct transfer from the ED to the OR and critical procedures in the ED. Moreover, in this study, we compared the red code group to the yellow code group and identified significant differences in indicators of injury severity and resource consumption. Given the absence of a gold standard for assessing triage tool accuracy, we believe that comparing these two groups more accurately reflects the triage tool’s ability to distinguish patients with and without major trauma. An additional limitation of this study is the small number of patients in the undertriage group, which prevented us from conducting statistical tests on this group. However, we seek to accomplish this in future studies.

## Conclusions

The two-tiered TTA system implemented in our institution enabled us to effectively distinguish between major trauma and minor trauma patients. Our key findings contribute to existing evidence supporting the efficiency and cost-effectiveness of a two-tiered TTA system. The results of this study may have implications for how trauma centers triage their patients and optimize resource utilization.

## Data Availability

No datasets were generated or analysed during the current study.
